# Is conservative management safe in patients with acute ureterolithiasis and perirenal stranding?

**DOI:** 10.1007/s00240-023-01411-z

**Published:** 2023-02-22

**Authors:** Nico C. Grossmann, Davide Ardizzone, Thomas Hermanns, Etienne X. Keller, Christian D. Fankhauser

**Affiliations:** Department of Urology, University Hospital Zurich, Frauenklinikstr. 10, 8091 Zurich, Switzerland

**Keywords:** Fornix rupture, Calyx rupture, Perirenal abscess, Ureteral stone, Perirenal stranding

## Abstract

In patients presenting with ureterolithiasis, perirenal stranding is frequently observed in non-contrast computed tomography. Because perirenal stranding may be caused by tears in the collecting system, previous studies have described an increased risk of infectious complications and suggested broad empiric antibiotic therapy and immediate decompressing of the upper urinary tract. We hypothesized that these patients can also be managed conservatively. Therefore, we retrospectively identified patients with ureterolithiasis and perirenal stranding and compared diagnostic and treatment characteristics as well as treatment outcomes between patients undergoing conservative versus interventional management by ureteral stenting, percutaneous drainage or primary ureteroscopic stone removal. We classified perirenal stranding as mild, moderate or severe based on its radiological extent. Of 211 patients, 98 were managed conservatively. Patients in the interventional group had larger ureteral stones, more proximal ureteral stone location, more severe perirenal stranding, higher systemic and urinary infectious parameters, higher creatinine levels, and received more frequent antibiotic therapy. The conservatively managed group experienced a spontaneous stone passage rate of 77%, while 23% required delayed intervention. In the interventional and conservative groups, 4% and 2% of patients, respectively, developed sepsis. None of the patients in either group developed a perirenal abscess. Comparison of perirenal stranding grade between mild, moderate and severe in the conservatively treated group showed no difference in the spontaneous stone passage and infectious complications. In conclusion, conservative management without prophylactic antibiotics for ureterolithiasis and perirenal stranding is a valid treatment option as long as no clinical or laboratory signs of renal failure or infections are observed.

## Introduction

Acute ureterolithiasis is one of the most common [1] urologic emergencies. If clinically suspected, non-contrast computed tomography (CT) represents an important diagnostic tool. On CT scans, aside from stone load, location, and size, perirenal fluid accumulation—referred as perirenal stranding—is seen in up to 50–80% of patients with acute ureterolithiasis [2–4]. Perirenal stranding in patients with ureteral obstruction is known to be a secondary sign of an increased pressure in the upper urinary system, but the pathogenesis of this phenomenon has not been fully clarified. It is believed that perirenal stranding may either be related to an increased pyelo-venous/lymphatic reflux with increasing pressure in the upper urinary tract or be a sign of tears in the collecting system and is often described as “fornix rupture” [2]. Because most patients are diagnosed by unenhanced CT, a true tear in the collecting system is rarely confirmed by a late contrast phase.

Current data on the clinical impact of perirenal stranding is sparse and conflicting. Previous studies have suggested an increased risk of urinoma, urinary tract infection and abscess when forniceal rupture is suspected [5], prompting the authors to recommend intervention and broad antibiotic coverage for such cases. Current international guidelines also recommended decompressing by the placement of a ureteral stent or a percutaneous nephrostomy tube if forniceal rupture is suspected [6]. On the other hand, patients with perirenal stranding have been adequately managed conservatively in previous series [2, 4, 7, 8]. The aim of this study was to investigate whether conservative management is safe in patients with ureteric stones with perirenal stranding and no signs of renal failure or urinary tract infection. In addition, we defined and compared three different grades of perirenal stranding.

## Patients and methods

We retrospectively reviewed data from a consecutive cohort of patients who presented at a tertiary care emergency department due to symptomatic ureterolithiasis between 2011 and 2017. All patients with radiologically detected perirenal stranding and ipsilateral ureterolithiasis were included. Exclusion criteria were anatomic aberrations, age under 18 years, missing laboratory values and missing follow-up of at least 30 days after admission for patients who were managed conservatively.

**Fig. 1:**
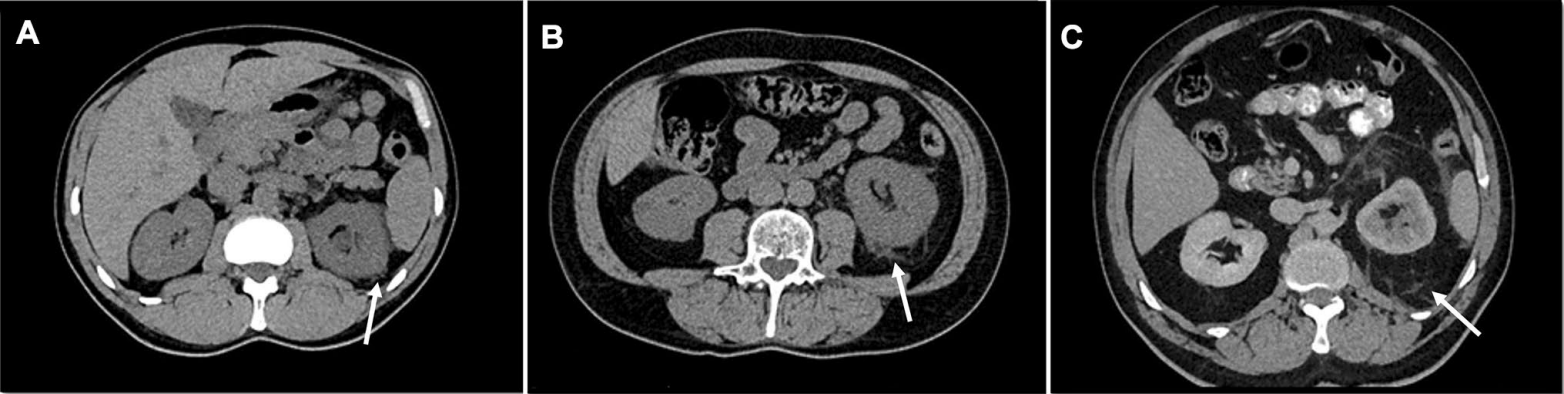
Examples of computed tomography scans with **A** mild, **B** moderate, and **C** severe perirenal stranding indicated by the white arrow

The review of the patient cohort included patient’s age; gender; vital signs on admission; laboratory values on admission, including blood and urine analysis; and radiological findings on admission, such as perirenal stranding, stone size—defined as the maximal stone diameter in the axial plane on CT-scan, and stone position. Moreover, we assessed not only treatment characteristics, including the use of empiric antibiotics and the type of surgical intervention, but also treatment outcomes such as the need for delayed surgical treatment in patients initially treated with a conservative approach, time to spontaneous stone passage in conservatively managed patients, development of sepsis—defined as the presence of at least two of the following host systemic inflammatory syndrome criteria [9]: tachycardia (heart rate > 90/min), tachypnea (respiratory rate > 20/min or PaCO2 < 32 mm Hg [4.3 kPa]), fever or hypothermia (temperature > 38 °C or < 36 °C), and leukocyte count > 12 G/l or < 4 G/l or > 10% immature bands—bacteraemia, need for intensive care unit and disease-related death. The severity of perirenal stranding was classified as mild, moderate or severe based on non-contrast-enhanced CT on admission, as described previously [2, 10]. Figure 1 illustrates the three different grades of perirenal stranding. Interventional management included ureteral stenting, primary ureterorenoscopic stone removal, shockwave lithotripsy or nephrostomy insertion within 24 h after admission. The decision to undergo interventional management was a shared decision with the patients and suggested in the presence of impaired renal function, refractory pain, suspected urinary tract infection or low probability of spontaneous stone passage according to stone location and size. All other patients were managed conservatively with clinical and sonographic follow-up, whose frequency was at the physicians’ discretion. Neither the presence nor the grade of perirenal stranding was an absolute indicator of interventional management, and its indication was at the physicians’ discretion. The primary objective of the study was to compare the treatment outcomes between patients with perirenal stranding who were treated by either interventional or conservative management. The secondary objective was to compare the outcomes in the conservatively treated group between the different grades of perirenal stranding.

We performed descriptive statistics for baseline characteristics and outcomes. Continuous normally distributed variables are expressed as mean ± standard deviation, while continuous non-normally distributed variables are presented as median with interquartile range (IQR), and categorical variables are presented as a percentage. We used Fisher’s exact test to assess associations between categorical variables, while the Mann–Whitney U test or *T* test was used to assess differences in continuous variables between the two treatment techniques. We also employed the Freeman-Halton extension to assess associations of categorical variables between the three perirenal stranding grade groups, and the Kruskal–Wallis test and an ANOVA were used to assess continuous variables between perirenal stranding grade groups. A *p*-value of less than 0.05 (two sided) was considered to be statistically significant. We performed statistical analyses using Microsoft Excel 2017 (Microsoft Corporation, Redmond, Washington, USA) and SPSS ^®^ Version 26 (IBM, Armonk, New York, USA). The study was approved by the local ethics committee (BASEC-Nr. 2017–02036).

## Results

Of 211 patients with perirenal stranding, 98 (46%) were treated conservatively (conservative management group), and 113 (54%) were managed by the intervention (interventional management group). Table 1 summarises the baseline characteristics, vital signs and laboratory values of all included patients, stratified by management type. Compared with patients managed conservatively, those managed by interventional therapy had larger stones, a higher proportion of proximal ureteral stones and a higher grade of perirenal stranding, and they were more often treated with an empiric antibiotic. Furthermore, patients managed by intervention had a lower diastolic blood pressure, a higher body temperature, lower thrombocyte counts, higher serum neutrophile counts, lower lymphocyte counts, higher serum urea levels, higher c-reactive protein (CRP), higher serum creatinine, higher urine leukocyte count and a higher proportion of positive urine culture than conservatively managed patients. In the interventional management group, 82 (73%) patients were treated by double J-catheter insertion, one (1%) patient by percutaneous drain insertion, six (5%) patients by primary ureteroscopic stone removal and 24 (21%) patients by shock wave lithotripsy.

**Table 1:** Baseline, treatment, vital and laboratory characteristics of patients with perirenal stranding either treated with interventional or conservative management

	Total cohort	Interventional management	Conservative management	*p*-value
Patient characteristics				
Number of patients (*n*, %)	211 (100)	113 (53.6)	98 (46.4)	
Female (*n*, %)	32 (15.2)	18 (15.9)	14 (14.3)	***0.8
Age (Median, IQR)	50 (41–59)	51 (41–62)	49 (41–57)	*0.19
Ureteral stone size [mm] (median, IQR)	5 (4–7)	7 (5–8)	4 (3–5)	* < 0.001
Position ureter stone (*n*, %)				*** < 0.001
Proximal ureter	62 (29.4)	50 (44.2)	12 (12.2)	
Middle ureter	38 (18.0)	23 (20.4)	15 (15.3)	
Distal ureter	111 (52.6)	40 (35.4)	71 (72.5)	
Stranding (*n*,%)				***0.8
Perirenal alone	157 (74.4)	83 (73.5)	74 (75.5)	
Perirenal and periureteral	54 (25.6)	30 (26.5)	24 (24.5)	
Periureteral alone	0 (0.0)	0 (0.0)	0 (0.0)	
Grade of stranding (*n*, %)				***0.03
Mild	102 (48.3)	45 (39.8)	57 (58.1)	
Moderate	71 (33.7)	44 (39.0)	27 (27.6)	
Severe	38 (18.0)	24 (21.2)	14 (14.3)	
Empiric antibiotic treatment (*n*,%)	42 (19.9)	37 (32.7)	5 (5.1)	*** < 0.001
Type of immediate intervention (*n*,%)				
Double J-catheter	–	82 (72.6)	–	
Percutaneous drain	–	1 (0.9)	–	
Primary ureteroscopic stone removal	–	6 (5.3)	–	
Shock wave lithotripsy	–	24 (21.2)	–	
Vital signs at admission				
Heart rate [min^−1^] (median, IQR)	74 (66–83)	73 (66–86)	74 (67–80)	*0.6
Blood pressure systolic [mmHg] (mean, SD)	152 (73)	155 (95)	149 (19)	**0.6
Blood pressure diastolic [mmHg] (mean, SD)	88 (16)	86 (18)	92 (13)	**0.005
Temperature [°C] (mean, SD)	36.8 (0.6)	36.9 (0.6)	36.6 (0.5)	** < 0.001
Laboratory values at admission				
Thrombocytes [G/l] (mean, SD)	242 (71)	233 (74)	252 (66)	**0.046
Leukocytes [G/l] (mean, SD)	11.5 (4.4)	11.8 (5.2)	11.0 (3.3)	**0.2
Neutrophiles [G/l] (mean, SD)	8.5 (4.7)	9.2 (5.5)	7.7 (3.5)	**0.04
Lymphocytes [G/l] (mean, SD)	2.0 (1.0)	1.8 (0.8)	2.2 (1.1)	**0.002
Urea [mmol/l] (mean, SD)	6.6 (2.3)	7.0 (2.8)	6.1 (1.4)	**0.02
CRP [mg/l] (mean, SD)	15 (43)	25 (56)	3.5 (9)	** < 0.001
Creatinine [µmol/l] (mean, SD)	108 (50)	118 (64)	97 (19)	**0.003
Leukocyturia [/µl] (mean, SD)	31 (88)	50 (117)	11 (13)	**0.002
Nitrite positive (*n*, %)	3 (1.4)	2 (1.8)	1 (1.0)	***0.8
Positive urine culture (> 10^4^/ml) (n, %)	50 (23.7)	36 (31.9)	14 (14.3)	***0.003

Bold values indicate a statistically significant difference (*p* < 0.05)

^*^Mann–Whitney U Test ** t test ***Fisher’s Exact Test

Table 2 displays the treatment outcomes of all patients, stratified by management type. While there were no spontaneous stone passages after interventional management, 75 conservatively treated patients (77%) experienced a stone passage after a median duration of four days (IQR 1–8). While all conservatively managed patients were treated in an outpatient setting, those managed by intervention had a median inpatient stay of three days (IQR 2–4). A delayed surgical treatment in the conservative management group was necessary for 23 patients (24%) due to persistent ureterolithiasis (*n* = 6), urinary tract infection (*n* = 3), renal failure (*n* = 1) and refractory pain (*n* = 13). Patients in the interventional group showed higher rates of sepsis, bacteraemia, need for intensive care unit admission and death compared with the conservative group, but the differences were not statistically significant. None of the patients in either group developed a renal abscess.

**Table 2:** Treatment outcomes of patients with perirenal stranding either treated with interventional or conservative management

	Total cohort	Interventional management	Conservative management	*p*-value
Outcome				
Median follow-up (days, IQR)	55 (32–192)	66 (41–253)	43 (18–135)	–
Spontaneous stone passage (n,%)	75 (35.5)	–	75 (76.5)	–
Days until stone passage (median, IQR)	4 (1–8)	–	4 (1–8)	–
Days of inpatient stay (median, IQR)	2 (0–3)	3 (2–4)	0 (0)	* < 0.001
Delayed interventional treatment (*n*, %)	–	–	23 (23.5)	–
Secondary ureteroscopic stone removal (after initial Double J-catheter-insertion)	–	–	12 (12.3)	–
Primary ureteroscopic stone removal	–	–	7 (7.1)	–
Shock wave lithotripsy	–	–	4 (4.1)	–
Sepsis (*n*, %)	6 (2.8)	4 (3.5)	2 (2.0)	**0.7
Bacteremia/positive blood cultures (*n*, %)	3 (1.4)	2 (1.8)	1 (1.0)	** > 0.9
Perirenal abscess (*n*, %)	0 (0)	0 (0)	0 (0)	
Transfer to ICU (*n*, %)	3 (1.4)	2 (1.8)	1 (1.0)	** > 0.9
Death within 30d after admission (*n*, %)	1 (0.5)	1 (0.9)	0 (0.0)	** > 0.9

Bold values indicate a statistically significant difference (*p* < 0.05)

^*^Mann–Whitney U Test, **Fisher’s Exact Test

To evaluate whether the grade of perirenal stranding has an impact on conservative treatment outcomes, we compared patients with mild, moderate and severe perirenal stranding within the conservative management group. Table 3 displays the baseline characteristics, vital signs, laboratory values and treatment outcomes of these patients, stratified by the grade of perirenal stranding. Patients with mild perirenal stranding were significantly younger than those with moderate and severe perirenal stranding. Moreover, patients with increasing perirenal stranding had higher systolic and diastolic blood pressure. Lymphocyte counts were significantly different between groups, while there was no difference in leukocyturia, blood leukocyte count, CRP and creatinine levels. We also observed no differences in stone size and location or body temperature between the three groups.

**Table 3:** Baseline, vital, laboratory and outcome characteristics of 98 conservative treated patients stratified by the grade of stranding

	Mild stranding	Moderate stranding	Severe stranding	*p *value
Patient characteristics				
Number of patients (*n*)	57	27	14	
Female gender (*n*, %)	9 (15.8)	4 (14.8)	1 (7.1)	***0.8
Age (Median, IQR)	45 (36–51)	55 (45–66)	56 (53–61)	* < 0.001
Ureteral stone size [mm] (median, SD)	4 (1.1)	4.0 (1.2)	4.5 (1.3)	*0.15
Position ureter stone* (n, %)*				***0.8
Proximal ureter	8 (14.0)	2 (7.4)	2 (14.3)	
Middle ureter	10 (17.6)	3 (11.1)	2 (14.3)	
Distal ureter	39 (68.4)	22 (81.5)	10 (71.4)	
Stranding* (n,%)*				*** > 0.9
Perirenal	57 (100.0)	27 (100.0)	14 (100.0)	
Periureteral^1^	14 (24.6)	7 (25.9)	3 (21.4)	
Empiric antibiotic treatment (n,%)	3 (5.3)	1 (3.7)	1 (7.1)	*** > 0.9
Vital signs				
Heart rate [min^−1^] (median, IQR)	77 (68–82)	71 (64–79)	73 (68–76)	*0.2
Blood pressure systolic [mmHg] (mean, SD)	146 (19)	147 (15)	162 (19)	**0.02
Blood pressure diastolic [mmHg] (mean, SD)	91 (13)	90 (12)	101 (13)	**0.04
Temperature [°C] (mean, SD)	36.5 (0.5)	36.8 (0.4)	36.5 (0.4)	**0.06
Laboratory values				
Thrombocytes [G/l] (mean, SD)	258 (69)	232 (53)	264 (70)	**0.2
Leukocytes [G/l] (mean, SD)	11.0 (3.3)	11.0 (2.8)	11.8 (4.0)	**0.6
Neutrophils [G/l] (mean, SD)	7.2 (3.5)	8.4 (2.9)	8.6 (4.0)	**0.3
Lymphocytes [G/l] (mean, SD)	2.46 (1.1)	1.78 (0.8)	2.19 (1.26)	**0.04
Urea [mmol/l] (mean, SD)	5.9 (1.4)	6.1 (1.4)	6.6 (1.4)	**0.5
CRP [mg/l] (mean, SD)	3 (5)	5 (16)	2 (1.7)	**0.5
Creatinine [µmol/l] (mean, SD)	92 (19)	101 (18)	104 (24)	**0.06
Leukocyturia [/µl] (mean, SD)	11 (13)	9 (6)	15 (24)	**0.4
Nitrite positive (*n*, %)	1 (1.8)	0 (0.0)	0 (0.0)	*** > 0.9
Positive urine culture (*n*, %)	9 (15.8)	3 (11.1)	2 (14.3)	*** > 0.9
Outcome				
Delayed interventional treatment (*n*, %)	15 (26.3)	7 (25.9)	1 (7.1)	*** > 0.9
Secondary ureteroscopic stone removal (after initial DJ-insertion)	7 (12.3)	4 (14.8)	1 (7.1)	
Percutaneous drain	0 (0.0)	0 (0.0)	0 (0.0)	
Primary ureteroscopic stone removal	5 (8.8)	2 (7.4)	0 (0.0)	
Shock wave lithotripsy	3 (5.2)	1 (3.7)	0 (0.0)	
Spontaneous stone passage (*n*, %)	42 (73.7)	20 (74.1)	13 (92.9)	***0.4
Days until stone passage (median, IQR)	4 (2–9)	5 (2–10)	4 (1–9)	* > 0.9
Sepsis (*n*, %)	1 (1.8)	1 (3.7)	0 (0)	***0.7
Bacteremia/positive blood cultures (n,%)	0 (0)	1 (3.7)	0 (0)	***0.4
Perirenal abscess (*n*, %)	0 (0)	0 (0)	0 (0)	
Transfer to ICU (*n*, %)	1 (1.8)	0 (0)	0 (0)	*** > 0.9
Death within 30d after admission (*n*, %)	0 (0)	0 (0)	0 (0)	-

Bold values indicate a statistically significant difference (*p* < 0.05)

^1^All patients with periureteral stranding did also show perirenal stranding

^*^Kruskal–Wallis Test, **ANOVA, ***Fisher’s Exact Test (Freeman-Halton extension)

Whereas delayed intervention was necessary for only one patient (7%) in the severe perirenal stranding group, it was necessary for 15 (26%) and seven (26%) in the mild and moderate perirenal stranding groups, although no statistically significant difference was observed (*p* > 0.9). Most patients (74–93%) experienced spontaneous stone passage after a median time of four to five days in all three groups.

Two patients from the conservative treatment group developed sepsis. One was a 21-year-old female patient who initially presented with a 4-mm proximal ureterolithiasis with mild perirenal stranding and inconspicuous blood and urine infection markers. Following initial conservative management, the patient was readmitted three days later due to a deteriorated general condition, fever up to 38 °C and chills. After the insertion of a ureteral stent, the patient had to be transferred to the intensive care unit with antibiotic therapy due to circulatory failure with hypotonic blood pressure and tachycardia. After eight days, she could be discharged in a good condition. The second patient was a 66-year-old female with an initial presentation with a 4-mm distal ureterolithiasis and moderate perirenal stranding. Aside from an elevated leucocytosis of 14 G/l, blood and urine infection parameters were within normal limits. One day after discharge from the emergency department with analgesics, she was readmitted with chills and a fever up to 38.3 °C. Blood cultures revealed growth of Proteus mirabilis. Intensive care was not necessary, and the patient could be discharged five days later after the insertion of a ureteral stent and antibiotic therapy. None of the patients in any of the groups died within 30 days of initial admission.

## Discussion

Perirenal stranding on non-contrasted CT can be observed as a secondary radiological sign mainly in patients with pyelonephritis or ureteral obstruction [11]. While perirenal stranding in patients with pyelonephritis is associated with fever and elevated laboratory inflammatory markers, the role in patients with ureteral obstruction without inflammatory markers is ambiguous [12]. Perirenal stranding in this setting results from increased upper urinary tract pressure by pyelo-venous/lymphatic reflux, whereby an incipient infection cannot be clearly excluded [2, 12]. Thus, several previous studies investigated the association of perirenal stranding with urinary tract infections and the impact on outcomes after different therapeutic approaches. Indeed, studies reported conflicting findings about the association of perirenal stranding with the risk of concomitant urinary tract infection [12, 13]. Authors reported an increased risk of infection after ureterorenoscopic stone removal or after ureteral stent insertion in patients with ureteral stone and perirenal stranding [14, 15]. In contrast, small retrospective studies reported successful conservative therapy in patients with spontaneously passable stones and the absence of clinical and laboratory signs of a urinary tract infection [7, 8]. Since studies have shown that perirenal stranding is mainly caused by small, distal stones, a conservative approach of these patients is desirable [7, 8, 16]. To the best of our knowledge, the present study includes the currently largest and most detailed cohort of patients with ureterolithiasis and concomitant perirenal stranding in whom the outcome was compared between conservative and interventional treatment.

In this cohort of 211 ureterolithiasis patients with perirenal stranding on CT, 98 patients (46%) were managed conservatively—93 thereof (95%) without any antibiotics. In this conservative management group, there was a high proportion of spontaneous stone passage within less than a week, as well as a low proportion of patient readmissions and infectious complications, with not a single abscess formation. These results are in line with two previously published cohorts of 40 and 103 patients, respectively [7, 8]. In our cohort, only 5% of the conservatively treated patients received empiric antibiotic therapy, suggesting that a higher proportion of patients with ureteric stone and perirenal stranding may be omitted from antibiotic treatment, and we observed infectious complications in only 2% of conservatively managed patients, which is contradictory to previous findings [5, 16, 17]. Considering the current literature, two new findings could be identified in the present study:

The first new finding of our study was a spontaneous stone passage in four out of five patients with a ureteric stone and perirenal stranding, which further supports a conservative management strategy in patients with small stones without impaired renal function, refractory pain, or suspected urinary tract infection. Especially since previous studies described an increased risk of infection after delayed/elective ureterorenoscopic stone removal in patients with perirenal stranding in their initial CT, emphasizes that a conservative management of these patients might be justified [14, 15]. However, it must be noted that ureteral obstruction may cause urine stasis of the upper urinary tract and an incipient infection proximal to the obstruction might not be identified by blood and urine analysis. This could further promote urinary tract infection if the stone does not pass spontaneously over time. While decompression of the upper urinary tract via ureteral stenting or nephrostomy is the treatment of choice for initial concomitant infection, early ureterorenoscopic stone removal could be performed in patients with impaired renal function or refractory pain instead of primary decompression followed by secondary stone removal. Particularly in the case of small distal stones, which is a predisposing factor for concomitant perirenal stranding, an early ureterorenoscopic stone removal seems to be accompanied by only few infections and complications [18, 19].

The second novel finding in this cohort was that even patients with more pronounced perirenal stranding could successfully be treated conservatively, as we found no difference in the rate of delayed intervention, spontaneous stone passing, abscess, sepsis or need for intensive care unit transfer. Thus, the extent of perirenal stranding does not seem to influence treatment outcomes. This could be explained by the theory of the perirenal stranding aetiology: Urinary reflux compensates for unilateral ureteral obstruction by creating increased pressure in the intrarenal collecting system to lower the overall upper urinary tract pressure. This results in decreased renal blood flow as the renovascular resistance increases [20, 21]. Consequently, urine production in the affected kidney slowly decreases; however, this mechanism requires time for the kidney to adjust to this condition. This mechanism may not occur immediately in the case of a sudden acute obstruction (e.g., one caused by a ureteral stone), and urine production continues, which further increases the intrapelvic renal pressure, leading to a reno-protective fornix rupture [22, 23]. Hence, it might be postulated that in the further clinical course, the kidney adapts to the given pressure conditions in the intrarenal collecting system and thus reduces the progression of fornix rupture. Therefore, the extent of perirenal stranding should not lead to an intervention per se.

Our study has limitations. First and foremost are the limitations associated with any retrospective data collection. For example, laboratory parameters could be influenced by existing comorbidities, and patients could have received unreported antibiotic treatment before evaluation. Additionally, no hard criteria were defined for the management decision, which was at the physician’s discretion and a shared decision with the patients, and consequently could have introduced confounders, nor was the follow-up of the patients standardised. Second, the small single-centre cohort allowed for only a purely descriptive analysis, such that no analyses with adjustments for possible confounders were possible. Moreover, patients were treated at a single academic centre, and our results may not be generalisable to other centres or populations.

## Conclusion

Conservative management without prophylactic antibiotics in the presence of ureteric stone with perirenal stranding on CT-scan seems to be a safe and effective treatment option for a majority of patients, as long as no clinical or laboratory signs of renal failure or infections are observed at the time of initial shared-decision making.

## Data Availability

Available upon request to the corresponding author.

## Code availability

Available upon request to the corresponding author.
